# Transcriptome characterisation of *Pinus tabuliformis* and evolution of genes in the *Pinus* phylogeny

**DOI:** 10.1186/1471-2164-14-263

**Published:** 2013-04-18

**Authors:** Shi-Hui Niu, Zhe-Xin Li, Hu-Wei Yuan, Xiao-Yang Chen, Yue Li, Wei Li

**Affiliations:** 1 National Engineering Laboratory for Forest Tree Breeding, Key Laboratory for Genetics and Breeding of Forest Trees and Ornamental Plants of the Ministry of Education, College of Biological Science and Technology, Beijing Forestry University, Beijing, 100083, People's Republic of China; 2 Laboratory of Bio-technology of Tropical and Subtropical Forestry, College of Forestry, South China Agriculture University, Guangzhou, 510642, People's Republic of China

**Keywords:** *Pinus tabuliformis* Carr, 454 pyrosequencing, SNPs, SSRs, *Pinus* phylogeny, Comparative transcriptomics

## Abstract

**Background:**

The Chinese pine (*Pinus tabuliformis*) is an indigenous conifer species in northern China but is relatively underdeveloped as a genomic resource; thus, limiting gene discovery and breeding. Large-scale transcriptome data were obtained using a next-generation sequencing platform to compensate for the lack of *P. tabuliformis* genomic information.

**Results:**

The increasing amount of transcriptome data on *Pinus* provides an excellent resource for multi-gene phylogenetic analysis and studies on how conserved genes and functions are maintained in the face of species divergence. The first *P. tabuliformis* transcriptome from a normalised cDNA library of multiple tissues and individuals was sequenced in a full 454 GS-FLX run, producing 911,302 sequencing reads. The high quality overlapping expressed sequence tags (ESTs) were assembled into 46,584 putative transcripts, and more than 700 SSRs and 92,000 SNPs/InDels were characterised. Comparative analysis of the transcriptome of six conifer species yielded 191 orthologues, from which we inferred a phylogenetic tree, evolutionary patterns and calculated rates of gene diversion. We also identified 938 fast evolving sequences that may be useful for identifying genes that perhaps evolved in response to positive selection and might be responsible for speciation in the *Pinus* lineage.

**Conclusions:**

A large collection of high-quality ESTs was obtained, *de novo* assembled and characterised, which represents a dramatic expansion of the current transcript catalogues of *P. tabuliformis* and which will gradually be applied in breeding programs of *P. tabuliformis*. Furthermore, these data will facilitate future studies of the comparative genomics of *P. tabuliformis* and other related species.

## Background

Conifers are widely distributed globally as the largest and most diverse group of gymnosperms [1] that evolved independently from angiosperms >300 million years ago [2]. Modern conifers are divided into eight families including 68 genera and 630 species, which form an integral part of the economy in many parts of the world [3]. Chinese pine (*Pinus tabuliformis* Carr.) is a widespread indigenous conifer species and an economically and ecologically important hard pine in northern China [4,5]. Because of its irreplaceable economic development and environmental protection status, a genetic improvement program for *P. tabuliformis* was initiated in the 1970s, and considerable progress has been made in many basic physiological aspects [4]. The study of natural genetic variation in *P. tabuliformis* has traditionally been investigated using a common garden approach, whereas the pace of development of genomic resources has been slow, as only 288 *P. tabuliformis* entries are included in the NCBI database. Information regarding the genetic control of many important traits and fine-scale genetic variations is extremely limited, and more is needed given the renewed emphasis to accelerate the pace of *P. tabuliformis* breeding and shorten the breeding cycle.

Despite the economic and ecological importance of the genus *Pinus*, the progress of entire genome sequencing and associated marker development has been limited [6,7]. Huge genomes with highly heterozygous and large amounts of repetitive DNA elements are the major obstacles towards sequencing the genomes of all *Pinus* spp. [8,9]. The genome sizes of conifers are larger than those of most other plant species. The genome in all extant members of the genus *Pinus* is 18,000–40,000 Mbp [10]. In contrast, several representative genera of angiosperm trees have genome sizes of 540–2,000 Mb [1]. Therefore, researchers have focused on the transcribed part of the genome using dedicated technologies [6,7]. Transcriptome analysis and construction of large-scale expressed sequence tag (EST) collections in pines are a promising means of providing genomic resources [2,9,11], as this technique produces expressed sequence portions of chromosomes at a fraction of the cost of sequencing the complete genome [12]. It also facilitates the analysis of the transcribed part of the genome, which is not easy to predict from the entire genome [13]. Next-generation sequencing is a viable and favourable alternative to Sanger sequencing and provides researchers with a relatively rapid and affordable option for developing genomic resources in non-model organisms [14-16]. The Roche 454 massively parallel pyrosequencing platform, GS FLX Titanium, can generate one million reads with an average read length of 400 bases at 99.5% accuracy per run [17,18].

In addition to the discovery of new genes and investigations of gene expression, thousands of simple sequence repeats (SSRs), single nucleotide polymorphisms (SNPs) and insertions and deletions (Indels) have been detected in transcriptome data [6,19]. It is possible to use these genome-wide and abundant markers to develop very dense genetic maps that can be applied to conduct marker-assisted selection breeding programs [20].

Moreover, the increasing availability of transcriptome data represents an excellent resource for comparative genomic analysis. Although there has been much work on the chloroplast DNA sequences (cpDNA) and mitochondria DNA sequences (mtDNA), based on phylogenetic analysis of *Pinus*[21-23], less emphasis has been placed on multi-gene phylogenetic analysis and on determination of how conserved genes and functions are maintained despite species divergence.

In the current study, we used the Roche 454 GS-FLX Titanium pyrosequencing platform to obtain a comprehensive transcriptome of *P. tabuliformis* from normalised cDNA libraries of adult trees (xylem, phloem, vascular cambium, needles, cones and strobili). As a result, thousands of molecular markers were characterised. Evolutionary studies based on these data and other shared transcriptome data of five pine species and one spruce species were conducted. These data provide compelling new insights into the transcriptome of *P. tabuliformis* and evolution of genes in the *Pinus* phylogeny.

## Results

### Transcriptome sequencing and *de novo* assembly

**Figure 1:**
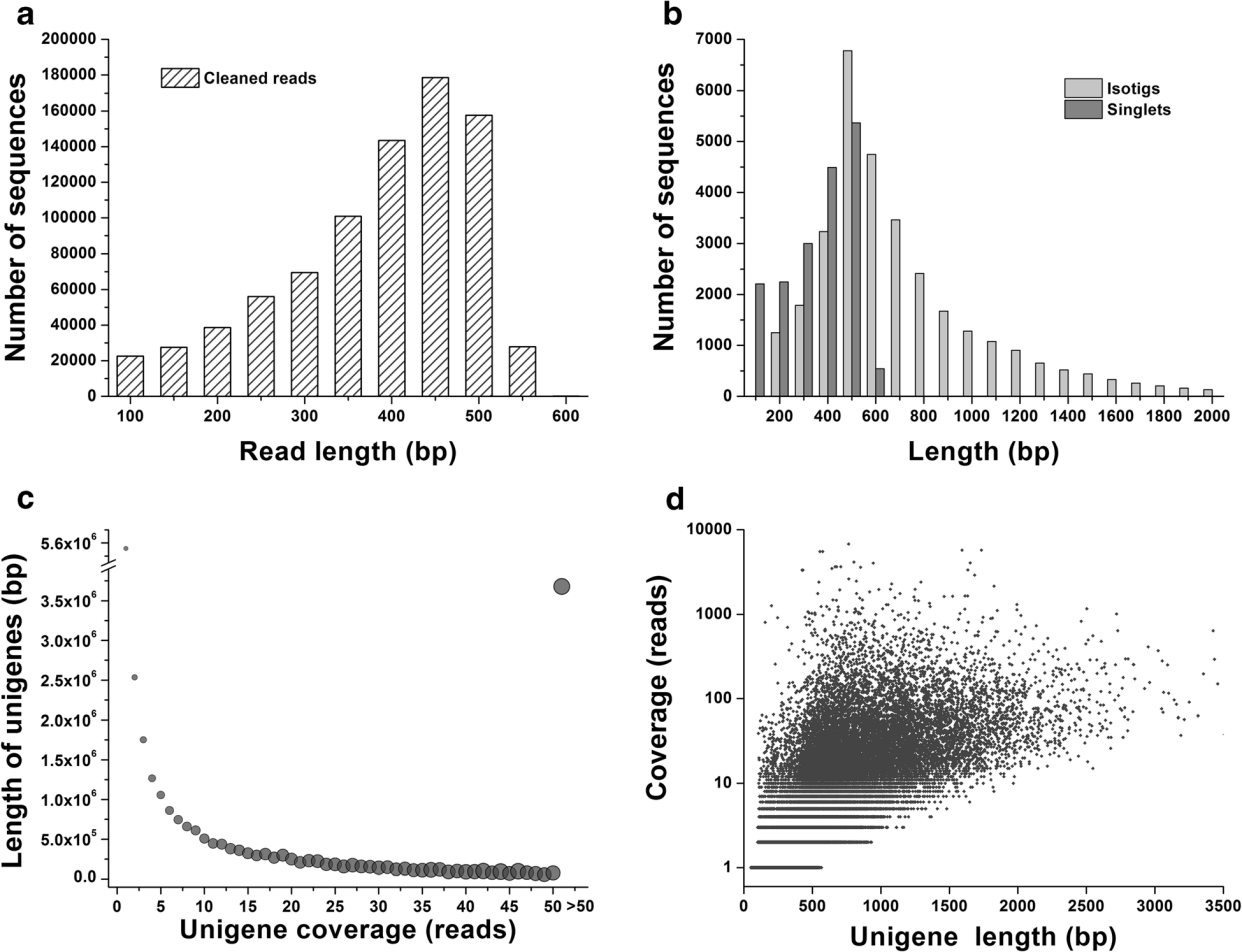
**Overview of *Pinus tabuliformis *transcriptome sequencing and assembly.** (**a**) Frequency distribution of 454 sequencing read lengths after filtering and trimming adapters. (**b**) Length distributions for isotigs and singlets following the *de novo* assembly process. The abscissa has been truncated at 2 kb. The longest isotig was 3,537 base pairs. An isotig is meant to be analogous to an individual transcript. (**c**) The average read-depth coverage for assembled unigenes. The y-axis label refers to the total length of all unigenes with the same read-depth coverage. Coverage values from 50 to 6,765 have been binned together. The size of the bubble is proportional to the average unigene length at the corresponding read-depth coverage. (**d**) A density scatter-plot showing the relationship between unigene length and coverage.

Prior to sequencing, the cDNA samples obtained from multiple tissues and individuals were normalised to increase the sequencing efficiency of rare transcripts. Subsequently, 911,302 raw reads with an average length of 382 bp were generated from a full 454 GS-FLX run. After a trimming process removed adaptors, primer sequences, poly-A tails as well as short, long and low quality sequences, 822,891 (84.7%) high-quality reads were obtained with an average length of 358 bp covering a total of 21,076,176 bases (Table 1, Figure 1a). Cleaned and qualified reads were *de novo* assembled using CAP3 and Newbler. This process produced a set of 31,623 isotigs and 17,853 remaining as singletons. More than half of the total assembly length of isotigs was > 700 bp (N50 = 744) (Table 1, Figure 1b).

**Table 1:** Sequencing, assembly and data analysis

**Raw results (after trimming)**		**Assembly results**	
Total number of reads	822 891	Total number of isotigs	31 623
Total read length (bp)	295 125 234	Total isotigs length (bp)	21 076 176
Minimum read length (bp)	50	Isotig N50 (bp)	744
Median read length (bp)	384	Maximum isotig length (bp)	3537
Maximum read length (bp)	578	Mean depth	28.2
Mean read length (bp)	358	Number of singletons	17 853
GC content (%)	43.2	Total number of unigenes	46 584

The unigene coverage distribution revealed that most unigenes had a read-depth coverage <20-fold (Figure 1c, d). The steep decline in read-depth coverage suggests that cDNA normalisation was effective, which is typical for a normalised library [24]. Isotig lengths were related to the number of sequences assembled into each isotig. The average unigene length exhibited a gradual increase with increasing read depth (Figure 1c, d).

### Functional annotation of the transcriptome

**Figure 2:**
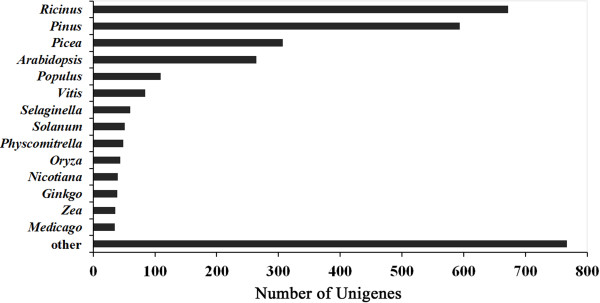
**Summary and taxonomic source of BLASTx matches to unigenes.** Number of unique best BLASTx matches of unigenes grouped by genus. The best matches of the unigenes to Pinaceae sequences accounted for 28.6% of the total.

**Figure 3:**
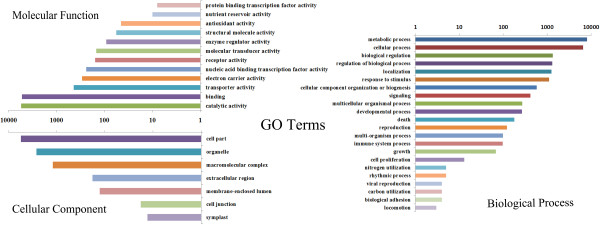
**Gene Ontology (GO) distributions for the *Pinus tabuliformis *transcriptome.** Main functional categories in the biological process, cellular component and molecular functions found in the transcriptome relevant to plant physiology. The abscissa indicates the number of unigenes. Bars represent the numbers of assignments of *Pinus tabuliformis* proteins with BLASTx matches to each GO term. One unigene may be matched to multiple GO terms.

The unigenes were annotated with gene names and Gene Ontology (GO) terms based on sequence comparisons between *P. tabuliformis* transcripts and the NCBI non-redundant protein database. We examined the taxonomic distribution of BLASTx best hits. As a result 99.3% (21,041) of the unigenes had a best hit to Pinaceae, but 95% were unknown functional proteins. Of the 3,151 genes with specific functional annotation, 18.9% were within *Pinus* and 9.7% were within *Picea* (Figure 2). The even distribution of assignments of proteins to more specialised GO terms further indicates that the *P. tabuliformis* 454 sequences represent proteins from a diverse range of functional classes (Figure 3).

### Identification of SSRs, SNPs and Indels

**Figure 4:**
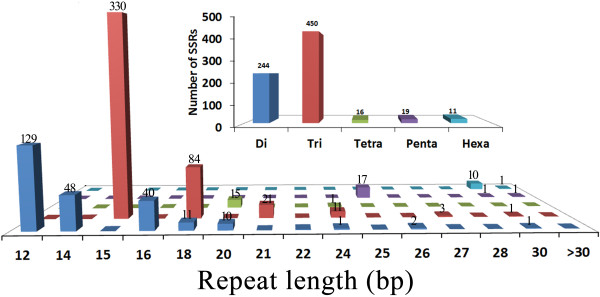
**Distribution of simple sequence repeats (SSRs) in *Pinus tabuliformis *expressed sequence tags (ESTs).** Di-, tri-, tetra-, penta- and hexa-nucleotide repeats were analysed and their frequencies plotted as a function of the repeat number. The upper right histogram shows the distribution of the total number of SSRs in different classes.

Di- to hexa-nucleotide SSRs with a minimum repeat unit size of five (for tri- to hexa-nucleotide) or six (for di-nucleotide) were identified based on the analysis of assembled isotig templates. A total of 724 distinct loci were identified, and the incidences of different repeat types were determined. The tri-nucleotide repeats were most abundant (62.2%), followed by di-nucleotides (33.7%), among the various classes of SSRs (Figure 4).

**Figure 5:**
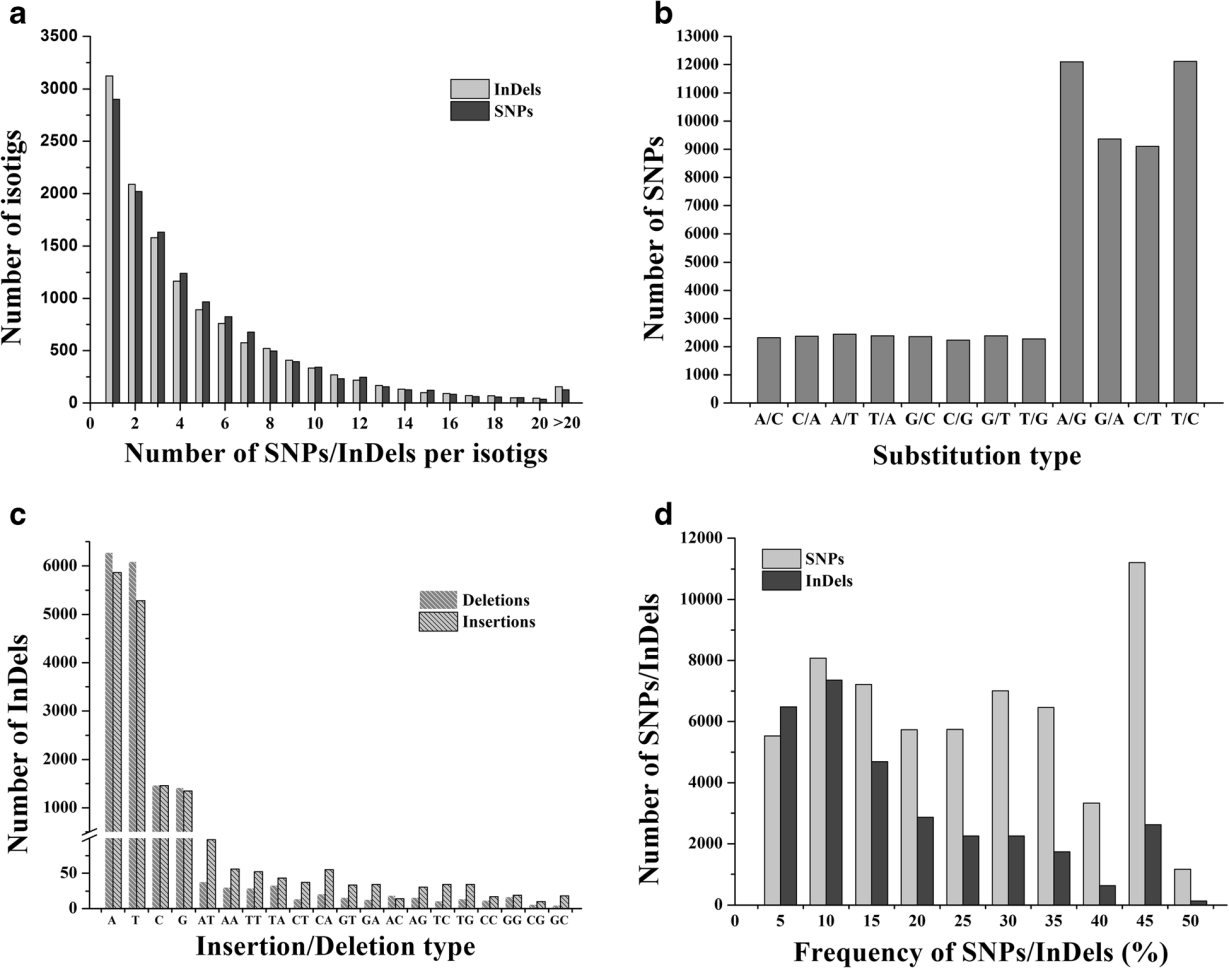
**Quality of single nucleotide polymorphisms (SNPs) and insertion/deletions (InDels) in *Pinus tabuliformis*** (**isotigs. a**) Numbers of SNPs and InDels detected per transcript. (**b**) Frequencies of different substitution types of SNPs. (**c**) Frequencies of different insertion/deletion types of InDels. (**d**) Distributions of SNPs and InDels in total transcripts. The x-axis represents the percentage of one SNP/InDel allele in the population.

More than 92,000 SNPs/Indels were identified (61,454 SNPs and 31,030 Indels) from the *P. tabuliformis* ESTs. The number of SNPs/Indels detected per transcript was highly variable; however, approximately 40% of the transcripts contained only one or two SNPs/Indels (Figure 5a). Among all SNPs, transitions (69.5%) were more frequent than transversions (30.5%) (Figure 5b). A and T were the most frequent insertion (76.7%) and deletion (79.5%) types of InDels (Figure 5c). The distribution of alternate allele frequencies in all contigs containing InDels was less frequent in total transcripts, but the SNPs were distributed evenly (Figure 5d).

### Orthologue identification and functional characterisation between six conifer species

Large-scale transcriptome characterisations have been carried out for *Pinus taeda*[3], *Pinus contorta*[25], *Pinus sylvestris*[26] and *Pinus pinaster*[2]. The shared transcriptomes of *Pinus* in the PlantGDB and NCBI databases are valuable sources of information for multi-gene comparative and phylogenetic analyses [27].

**Figure 6:**
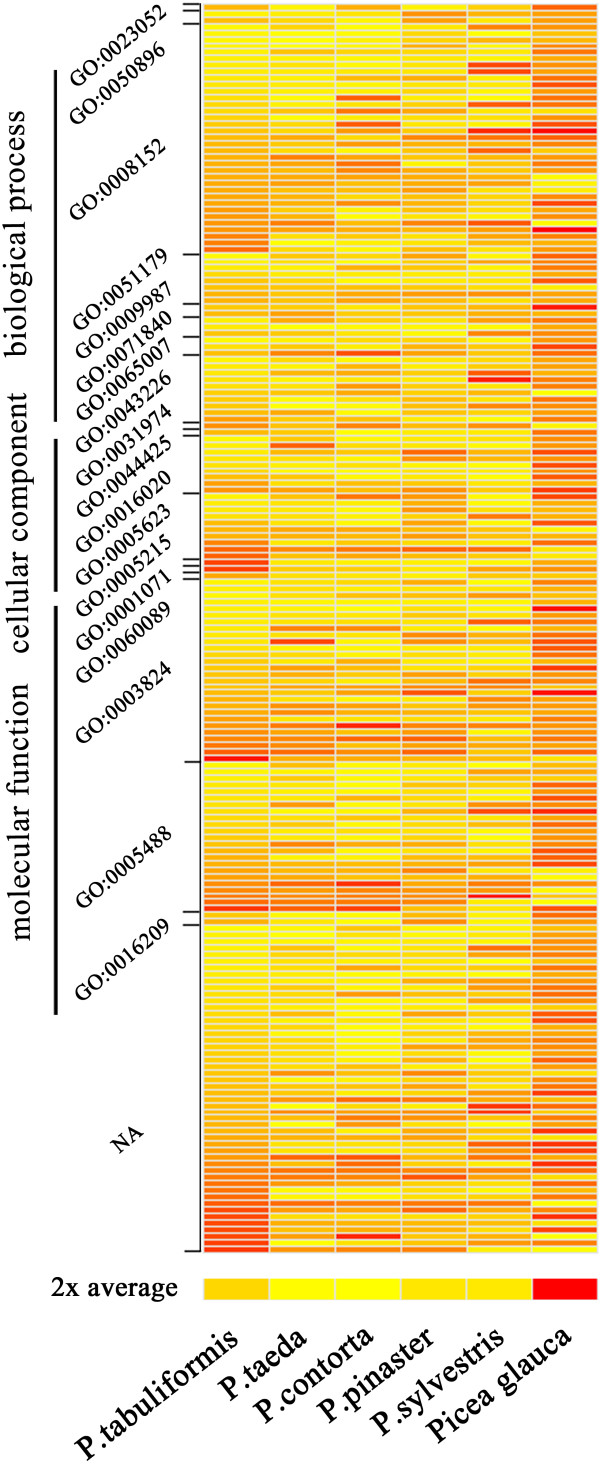
**Functional annotation and divergence between homologs of five pine and one spruce species.** The heat map is based on the 191 putatively orthologous transcripts of six species. The homologs were annotated with Gene Ontology (GO) terms. Colours indicate similarity from yellow (highly similar) to red (weakly similar). The “2× average” is an overall measure of how similar the different species are.

A comparative analysis of the transcriptomes of *P. tabuliformis*, *P. contorta*, *P. pinaster*, *P. sylvestris*, *P. taeda* and *Picea glauca* yielded 191 putatively orthologous sets of ESTs (Additional file 1). The orthologues were annotated with GO terms, and 54 orthologues were involved in biological processes, 33 orthologues were involved in cellular components, 54 orthologues were involved in molecular functions and the other 50 orthologues had unknown biological functions (Figure 6).

### Phylogenetic and speciation analysis

**Figure 7:**
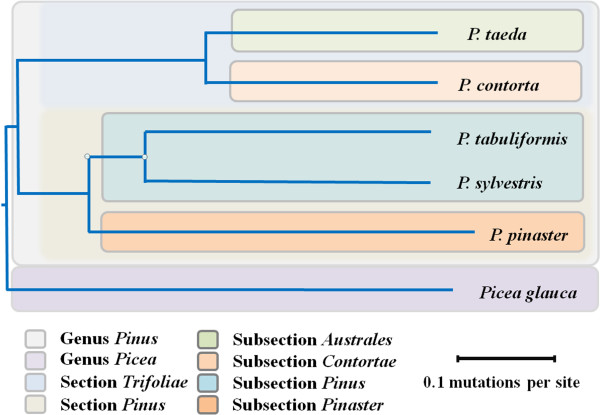
**Phylogram of the five pine and one spruce species.** Phylogram derived using pairwise non-synonymous substitution rates of orthologous transcripts as a distance metric (not from multiple sequence alignments) and the neighbour-joining method [66]. Branch lengths indicate the non-synonymous substitution rates between different species.

Phylogenetic analyses of *Pinus* species and *Picea glauca* as an out-group were conducted from 191 clusters of orthologous transcripts, using non-synonymous substitution rates as a distance metric. The results in Figure 7 show good agreement with classical taxonomy. Similar concordance was observed in the cpDNA and mtDNA-based reconstructions of the *Pinus* phylogeny [21,22].

**Figure 8:**
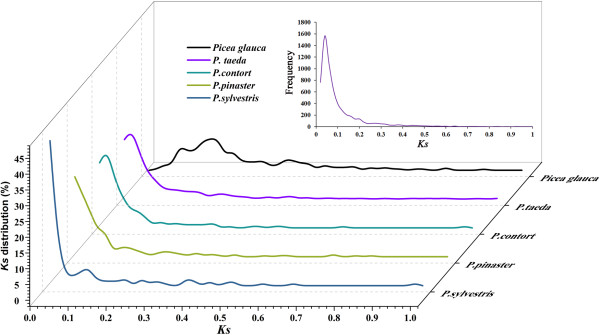
**Distribution of Ks values of orthologous pairs for identifying speciation events.** Data were grouped into bins of 0.02 *Ks* units for graphing. The upper right graph shows the *Ks* distribution of the 6,053 pairs of orthologues identified between *P. tabuliformis* and *P. taeda*. Given the rate of substitutions/synonymous site per year, the peaks (*Picea glauca* = 0.1, *P. taeda* and *P. contort* = 0.03, *P. pinaster* and *P. sylvestris* < 0.01) indicate the speciation time between *P. tabuliformis* and these species.

We estimated the level of synonymous substitutions for 191 pairs of orthologues identified among the six species and 6,053 pairs of orthologues identified between *P. tabuliformis* with *P. taeda* as a control to assess the relative age of these species separations. The *Ks* peaks (*Picea glauca* = 0.1, *P. taeda* and *P. contort* = 0.03, *P. pinaster* and *P. sylvestris* < 0.01) indicate the speciation time between *P. tabuliformis* and these species (Figure 8). Considering a clock-like synonymous mutation rate of 0.68 × 10^-9^ substitutions/site/year in conifer genes based on pairwise comparisons of 3,723 spruce (*Picea sitchensis*) and pine (*P. taeda*) orthologues [28], the speciation between spruce and pine was estimated to have occurred ~147 million years ago (mya) and between section *Trofoliae* and section *Pinus* ~44 mya ago.

### Evolutionary pattern of *Pinus* spp. genes

**Figure 9:**
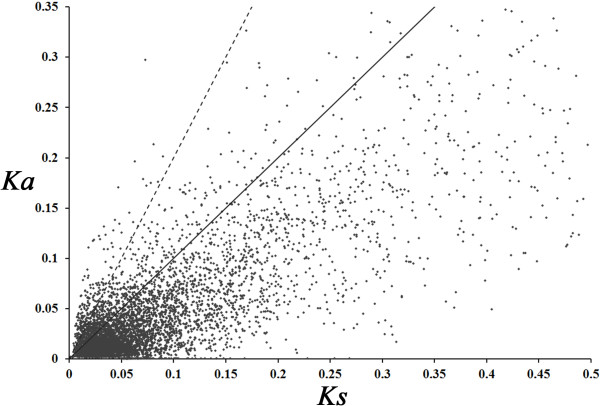
***Ka*/*Ks *distribution among 6053 homolog pairs of *Pinus tabuliformis *and *P. taeda*.** The mean Ka/Ks value was 0.63. The solid line shows the threshold of Ka/Ks = 1, whereas the dashed line marks the more conservative threshold of *Ka*/*Ks* = 2. Overall 938 orthologous sequences fell above the light solid line and 207 sequences fell above the dashed line.

We estimated evolutionary measures at 6,053 orthologues of *P. tabuliformis* and *P. taeda*. The number of pairwise synonymous (*Ks*) and non-synonymous (*Ka*) substitutions per site was inferred (Figure 9). The results show that a majority of sequence pairs (85%) had a *Ka*/*Ks* ratio < 1, suggesting that these evolved under purifying selection without altering the encoded amino acid sequence during the speciation period. We also identified 938 fast evolving sequences with a *Ka*/*Ks* ratio > 1 and 207 sequences with *Ka*/*Ks* ratios > 2 (Additional file 2). These sequences are related to several biological processes, cellular components and molecular functions. Therefore, these ESTs may be useful for identifying genes that may have evolved in response to positive selection and might be responsible for speciation in the *Pinus* lineage.

## Discussion

A large number of ESTs for pines have been sequenced to date (Table 2). Due to the economic value of wood and pulp products, the initial EST projects on pine focused primarily on the transcriptional regulation of wood formation [29]. Large numbers of ESTs have been sequenced and analysed in pine to discover wood formation and wood quality trait related genes [30-33]. Sequencing novel genes expressed during wood formation represents a powerful approach to understanding wood formation at the molecular level and identifying the mechanisms that control this important differentiation pathway. A total of 260 differentially expressed sequences have been identified across six cDNA libraries from the xylem of *P. taeda*[34]. A large number of represented gene sequences from xylem-forming tissues of loblolly pine have been compared with the inferred gene sequences of *Arabidopsis thaliana*[33]. In addition, 42 EST resembled gene products important for drought tolerance have been identified from root tissue libraries [35]. To study similarities between angiosperm and gymnosperm embryo development, 83 embryogenesis-related genes were identified from embryo cDNA libraries [3]. Most genomic studies of pines have focused on loblolly pine, while additional sequencing efforts are needed to develop genomic resources for other pines.

**Table 2:** **Transcriptome sequencing in *Pinus *spp.**

***Pinus *spp.**	**Platform or approach**	**Libraries**	**Reads or ESTs**	**Mean length**	**Unigenes**	**References**
*P. taeda*	cDNA clones	xylem	1 097	510	736	[32]
*P. taeda*	cDNA clones	xylem	59 797	364	20 377	[33]
*P. taeda*	cDNA clones	root	12 918	555	6 202	[35]
*P. taeda*	cDNA clones	embryos	68 721	689	12 154	[3]
*P. radiata*	cDNA clones	xylem	6 389	624	3 304	[29]
*P. contorta*	GS XL R70	needles and conelets	586 732	306	17 000	[9]
*P. pinaster*	Sanger and GS-FLX	different tissues	951 641	597	55 332	[2]
*P.densata*	Illumina	needles	3 968 794		84 950	[67]

Despite the fact that a large number of pine ESTs have been obtained from cDNA libraries based on traditional sequencing technology, the methods used were inefficient. Four *P. taeda* cDNA libraries were sequenced and yielded a total of 142,533 ESTs (Table 2); however, only one normalised cDNA library yielded 822,891 ESTs in *P. tabuliformis* (Table 1). Although traditional sequencing yields longer EST sequences, it has little advantage compared to new assembly technology based on the large-scale ESTs. Additionally, most previous studies used a cDNA library of one tissue (Table 2), whereas we used a normalised cDNA library comprising multiple tissues and individuals. These large-scale ESTs will provide more comprehensive pine transcriptome information and facilitate the assembly of *Pinus* spp. ESTs in the future.

Next-generation sequencing technology yields a large number of sequences at considerably lower costs compared to traditional sequencing methods, and, therefore, provides a valuable starting point to expedite analysis of less-studied species [18,36,37]. Normalised cDNA libraries were used to sample large numbers of transcripts to maximise sequence diversity. Next-generation sequencing of normalised libraries is more efficient than that of non-normalised libraries, particularly for rare transcripts [38]. The capacity to deliver large numbers of gene-based markers from transcriptome sequencing projects is a major advantage of next-generation sequencing technology [18,20,36]. Because of cost and throughput, conventional markers such as restriction fragment length polymorphism and random amplified polymorphic DNA are being replaced with SSRs and SNPs [20]. The genome-wide and abundant EST-based SSRs and SNPs/Indels markers obtained by next-generation sequencing represent an effective approach to marker discovery in many plant species, as these markers facilitate generation of dense genetic maps and have the advantage of higher cross-species transferability [6,39-41]. However, relevant studies in *Pinus* spp. are limited. In this study, 724 distinct EST-SSR loci and more than 92,000 SNPs/InDels were identified. It is possible to use these markers in a broad range of applications, including genetic mapping, genotype identification, marker-assisted selection breeding, and molecular tagging of genes. Among the EST-derived SSRs, tri-nucleotide repeat units were predominant. Considering the importance of maintaining reading frames to generating a polypeptide within a partially or fully active, it is no surprise that this observation is common for tri-nucleotide expansions (or their multiples) within translated regions [6,42,43]. As usual, comparisons of *P. tabuliformis* transcriptome SNPs show an excess of transitional over transversional substitutions. Similarly, A and T were the most frequent insertion and deletion types of Indels. Part of this bias is due to the relatively high rate of mutation of methylated cytosines to thymines [44,45].

Comparative phylogenetic analysis at the genome level dramatically improves the precision and sensitivity of evolutionary inference [46]. However, comparative genomics in plants has been limited by the considerable phylogenetic distances between sequenced organisms [47]. Transcriptome sequencing using massively parallel sequencing technologies provides an attractive approach to obtaining large-scale sequence data for non-model organisms necessary for comparative genomic analysis [24,48]. Phylogenetic utility of transcriptome sequence data yields well-resolved and highly supported tree topologies for many groups of animals [49-51]; however, few such studies have been conducted with plant taxa [27]. Phylogenetic analysis of the genus *Pinus* has been limited mostly to plastid genome (cpDNA and mtDNA) sequences [21-23]. The results of this study are consistent with previous data on plastid genome phylogeny [21,22], but transcriptome analyses, producing more robust results, are presented for the first time. Given that this study was not limited to particular genes or motifs, the results presented here are more representative of *Pinus* evolution than previous studies.

Understanding the factors that affect the evolutionary patterns and rates of genes is central in many research fields [52]. For the past 30 years, it was thought that the rate of gene evolution was determined by protein function [53]. Studies on yeast and bacteria indicate that the expression level of a protein affects the evolution rate more than its functional category, at least in unicellular species [54,55]. In this study, we have shown that sequence polymorphisms of the 191 putatively orthologous sets of ESTs of six *Pinus* species are widespread using GO terms. This suggests that selection of protein function does not contribute to the variation in the rates of gene evolution. However, most of the important factors are correlated with each other. More systematic analyses of genomic data are required to further demonstrate the effect of a range of factors on the evolutionary patterns and rates of genes.

## Conclusions

This study is the first comprehensive sequencing effort and analysis of gene function in the transcriptome of *P. tabuliformis* and represents the most extensive expressed sequence resource available for *P. tabuliformis* to date. GO and KEGG analyses were carried out, and all unigenes were classified into functional categories so as to understand their functions and regulation pathways. An enormous number of SSR and SNP/Indel loci were detected. These data can be used to develop oligonucleotide microarrays or serve as a reference transcriptome for future RNA-seq experiments in large-scale gene expression assays. These data will accelerate our understanding of genetic variation in populations and the genetic control of important traits in *P. tabuliformis*. Additionally, the generation of such large-scale sequence data is a potentially invaluable scientific resource for mapping, marker-assisted breeding and conservation-genetic-oriented studies in *P. tabuliformis* and comparative evolutionary analysis of *Pinus* plants.

## Methods

### Sample collection, cDNA library creation and 454 sequencing

*P. tabuliformis* tissues were collected from 4–20 individual trees selected at random (genetically distinct) in a primary clonal Chinese pine seed orchard located in Xingcheng City, Liaoning Province, China (40°44’N, 120°34’E, 100 m above sea level) [4]. The sampling time and number of individuals of each tissue type are listed in Table 3. Developing xylem tissues were scraped from the exposed xylem surface at breast height (1.5 m) after removing the bark from the sampling area. Samples were immediately placed in liquid nitrogen in the field until storage at −80°C.

**Table 3:** Samples used for sequencing

	**Samples in May**	**Number of individuals**	**Samples in July**	**Number of individuals**
Tissue type	Cones	10	Strobili	10
	Cambium (stress side)	4	Cambium (stress side)	4
	Cambium (tension side)	4	Cambium (tension side)	4
	Cambium (stem)	4	Cambium (stem)	4
	Needles (juvenile + mature)	20		

Total RNA isolation from samples of all selected plant tissues, and cDNA library construction and normalisation were performed as described previously [56]. The pooled library was sequenced in a full 454 plate run on the GS-FLX Titanium platform (Roche, Indianapolis, IN, USA).

### Assembly and annotation

All generated ESTs were pre-screened to remove adaptor-ligated regions and contaminants by Seqclean and to trim low-quality regions by LUCY2 [57]. Because no reference *P. tabuliformis* genome exists, cleaned and qualified reads were assembled *de novo* in Newbler 2.5.3, which performs best for restoring full-length transcripts [13,58].

The assembled isotig and singleton sequences were combined and clustered with CD-HIT (version 4.0) [59,60]. The sequences with similarity >95% were divided into one class, and the longest sequence of each class was treated as a unigene during later processing. Descriptive annotations and GO classifications were performed as described previously [56].

We simultaneously instituted a search for putative unigenes against the NCBI protein database using a BLASTx and annotated each sequence with GO terms using Blast2GO.

### Identification of SSRs, SNPs and InDels

Assembled isotigs with coverage of at least four reads were screened for SSRs, SNPs and InDels using Misa and ssahaSNP software, respectively [61]. Similar criteria for screening high-quality SNPs have been used in previous studies [20,62]. Only perfect repeats of two to six nucleotide repeats were identified. The minimum repeat-unit size for di-nucleotides was set at six and at five for tri- to hexa-nucleotide repeats.

### Identification of orthologues between six conifer species

The shared transcriptome data of five conifer species in the PlantGDB and NCBI databases were downloaded. The numbers of unigenes for each species were as follows: *Picea glauca* (48,619), *P. contorta* (13,570), *P. pinaster* (15,648), *P. sylvestris* (73,609) and *P. taeda* (77,540). Along with 46,584 unigenes of *P. tabuliformis*, clustering was carried out among the transcribed sequences using UCLUST software [63]. Aligned sequences (at least 100 bp) showing 90% identity were defined as pairs of putative orthologues among six species. The best-hit sequence of each cluster was then used in subsequent analyses. Orthologues of *P. taeda* and *P. tabuliformis* were searched using the same approach. Sequences of *P. tabuliformis* were annotated with GO terms using Blast2GO.

### Estimation at the level of synonymous substitution and non-synonymous substitution between orthologues

Because unigenes are derived from EST sequences, have no annotated open reading frames and may contain frame shift sequencing errors, each member of a pair of sequences was searched using BLASTX against all plant protein sequences available in GenBank. The approach used was as described previously [64]. PAML software was used to estimate the non-synonymous substitutions per non-synonymous site (*Ka*) and the synonymous substitutions per synonymous site (*Ks*) [65].

### Phylogenetic analysis

Because the genus *Pinus* has a rich history of phylogenetic analysis and the relationships among the species in the genus are well understood [21-23], the precise topology is not critical for the purposes of this study. We chose to focus our analyses on the evolutionary pattern and rate of genes. The synonymous substitution and non-synonymous substitution between the orthologues of six conifer species were analysed as described previously. Phylograms were derived using pairwise substitution rates of orthologous transcripts as a distance metric with the neighbour-joining method [66]. *Picea glauca* was used as an out-group to root trees.

### Data availability

The raw 454 EST data obtained in this study were deposited in the NCBI Sequence Read Archive (SRA) under the accession number SRA 056887.

## Competing interests

The authors declare that they have no competing interests.

## Authors’ contributions

SHN participated in the sequence alignment and drafted the manuscript. ZXL and HWY participated in the samples preparation and 454 sequencing. XYC and YL participated in the design of the study and performed the statistical analysis. WL conceived of the study, and participated in its design and coordination and helped to draft the manuscript. All authors read and approved the final manuscript.

## Supplementary Material

Additional file 1 The 191 orthologues of six conifer species. Click here for file

Additional file 2 **The 207 sequences with Ka/Ks ratios > 2 of *Pinus tabuliformis *and *P. taeda***. Click here for file

## References

[B1] Ahuja MR Neale DB Evolution of genome size in conifers Silvae Genet 2005 54 3 126 137

[B2] Fernandez-Pozo N Canales J Guerrero-Fernandez D Villalobos DP Diaz-Moreno SM Bautista R Flores-Monterroso A Guevara MA Perdiguero P Collada C EuroPineDB: a high-coverage web database for maritime pine transcriptome BMC Genomics 2011 12 366 10.1186/1471-2164-12-366 21762488 PMC3152544

[B3] Cairney J Zheng L Cowels A Hsiao J Zismann V Liu J Ouyang S Thibaud-Nissen F Hamilton J Childs K Expressed sequence tags from loblolly pine embryos reveal similarities with angiosperm embryogenesis Plant Mol Biol 2006 62 4–5 485 501 17001497 10.1007/s11103-006-9035-9

[B4] Li W Wang X Li Y Stability in and correlation between factors influencing genetic quality of seed lots in seed orchard of *Pinus tabuliformis* Carr. over a 12-year span PLoS One 2011 6 8 e23544 10.1371/journal.pone.0023544 21887269 PMC3160887

[B5] Chen K Abbott RJ Milne RI Tian XM Liu J Phylogeography of *Pinus tabulaeformis* Carr. (Pinaceae), a dominant species of coniferous forest in northern China Mol Ecol 2008 17 19 4276 4288 10.1111/j.1365-294X.2008.03911.x 19378405

[B6] Lesser MR Parchman TL Buerkle CA Cross-species transferability of SSR loci developed from transciptome sequencing in lodgepole pine Mol Ecol Resour 2012 12 3 448 455 10.1111/j.1755-0998.2011.03102.x 22171820

[B7] Ralph SG Yueh H Friedmann M Aeschliman D Zeznik JA Nelson CC Butterfield YS Kirkpatrick R Liu J Jones SJ Conifer defence against insects: microarray gene expression profiling of Sitka spruce (*Picea sitchensis*) induced by mechanical wounding or feeding by spruce budworms (*Choristoneura occidentalis*) or white pine weevils (*Pissodes strobi*) reveals large-scale changes of the host transcriptome Plant Cell Environ 2006 29 8 1545 1570 10.1111/j.1365-3040.2006.01532.x 16898017

[B8] Valledor L Jorrin JV Rodriguez JL Lenz C Meijon M Rodriguez R Canal MJ Combined proteomic and transcriptomic analysis identifies differentially expressed pathways associated to *Pinus radiata* needle maturation J Proteome Res 2010 9 8 3954 3979 10.1021/pr1001669 20509709

[B9] Parchman TL Geist KS Grahnen JA Benkman CW Buerkle CA Transcriptome sequencing in an ecologically important tree species: assembly, annotation, and marker discovery BMC Genomics 2010 11 180 10.1186/1471-2164-11-180 20233449 PMC2851599

[B10] Morse AM Peterson DG Islam-Faridi MN Smith KE Magbanua Z Garcia SA Kubisiak TL Amerson HV Carlson JE Nelson CD Evolution of genome size and complexity in *Pinus* PLoS One 2009 4 2 e4332 10.1371/journal.pone.0004332 19194510 PMC2633040

[B11] Neale DB Genomics to tree breeding and forest health Curr Opin Genet Dev 2007 17 6 539 544 10.1016/j.gde.2007.10.002 18060764

[B12] Emrich SJ Barbazuk WB Li L Schnable PS Gene discovery and annotation using LCM-454 transcriptome sequencing Genome Res 2007 17 1 69 73 17095711 10.1101/gr.5145806 PMC1716268

[B13] Mundry M Bornberg-Bauer E Sammeth M Feulner PG Evaluating characteristics of de novo assembly software on 454 transcriptome data: a simulation approach PLoS One 2012 7 2 e31410 10.1371/journal.pone.0031410 22384018 PMC3288049

[B14] Hiremath PJ Farmer A Cannon SB Woodward J Kudapa H Tuteja R Kumar A Bhanuprakash A Mulaosmanovic B Gujaria N Large-scale transcriptome analysis in chickpea (*Cicer arietinum* L.), an orphan legume crop of the semi-arid tropics of Asia and Africa Plant Biotechnol J 2011 9 8 922 931 10.1111/j.1467-7652.2011.00625.x 21615673 PMC3437486

[B15] Fraser BA Weadick CJ Janowitz I Rodd FH Hughes KA Sequencing and characterization of the guppy (*Poecilia reticulata*) transcriptome BMC Genomics 2011 12 202 10.1186/1471-2164-12-202 21507250 PMC3113783

[B16] Russell JR Bayer M Booth C Cardle L Hackett CA Hedley PE Jorgensen L Morris JA Brennan RM Identification, utilisation and mapping of novel transcriptome-based markers from blackcurrant (*Ribes nigrum*) BMC Plant Biol 2011 11 147 10.1186/1471-2229-11-147 22035129 PMC3217869

[B17] Wang Y Zeng X Iyer NJ Bryant DW Mockler TC Mahalingam R Exploring the switchgrass transcriptome using second-generation sequencing technology PLoS One 2012 7 3 e34225 10.1371/journal.pone.0034225 22479570 PMC3315583

[B18] Kaur S Pembleton LW Cogan NO Savin KW Leonforte T Paull J Materne M Forster JW Transcriptome sequencing of field pea and faba bean for discovery and validation of SSR genetic markers BMC Genomics 2012 13 104 10.1186/1471-2164-13-104 22433453 PMC3352077

[B19] Renaut S Nolte AW Bernatchez L Mining transcriptome sequences towards identifying adaptive single nucleotide polymorphisms in Lake Whitefish species pairs (*Coregonus* spp. Salmonidae) Mol Ecol 2010 19 Suppl 1 115 131 20331775 10.1111/j.1365-294X.2009.04477.x

[B20] Barbazuk WB Emrich SJ Chen HD Li L Schnable PS SNP discovery via 454 transcriptome sequencing Plant J 2007 51 5 910 918 10.1111/j.1365-313X.2007.03193.x 17662031 PMC2169515

[B21] Eckert AJ Hall BD Phylogeny, historical biogeography, and patterns of diversification for *Pinus* (Pinaceae): phylogenetic tests of fossil-based hypotheses Mol Phylogenet Evol 2006 40 1 166 182 10.1016/j.ympev.2006.03.009 16621612

[B22] Gernandt DS Lopez GG Garcia SO Liston A Phylogeny and classification of *Pinus* Taxon 2005 54 1 29 42 10.2307/25065300

[B23] Wang B Mao JF Gao J Zhao W Wang XR Colonization of the Tibetan Plateau by the homoploid hybrid pine *Pinus densata* Mol Ecol 2011 20 18 3796 3811 10.1111/j.1365-294X.2011.05157.x 21689188

[B24] Der JP Barker MS Wickett NJ DePamphilis CW Wolf PG De novo characterization of the gametophyte transcriptome in bracken fern, *Pteridium aquilinum* BMC Genomics 2011 12 99 10.1186/1471-2164-12-99 21303537 PMC3042945

[B25] DiGuistini S Ralph SG Lim YW Holt R Jones S Bohlmann J Breuil C Generation and annotation of lodgepole pine and oleoresin-induced expressed sequences from the blue-stain fungus *Ophiostoma clavigerum*, a Mountain Pine Beetle-associated pathogen FEMS Microbiol Lett 2007 267 2 151 158 10.1111/j.1574-6968.2006.00565.x 17328114

[B26] Heller G Adomas A Li G Osborne J van Zyl L Sederoff R Finlay RD Stenlid J Asiegbu FO Transcriptional analysis of *Pinus sylvestris* roots challenged with the ectomycorrhizal fungus *Laccaria bicolor* BMC Plant Biol 2008 8 19 10.1186/1471-2229-8-19 18298811 PMC2268937

[B27] Logacheva MD Kasianov AS Vinogradov DV Samigullin TH Gelfand MS Makeev VJ Penin AA De novo sequencing and characterization of floral transcriptome in two species of buckwheat (Fagopyrum) BMC Genomics 2011 12 30 10.1186/1471-2164-12-30 21232141 PMC3027159

[B28] Buschiazzo E Ritland C Bohlmann J Ritland K Slow but not low: genomic comparisons reveals slower evolutionary rate and higher *dN*/*dS* in conifers compared to angiosperms BMC Evol Biol 2012 12 1 8 10.1186/1471-2148-12-8 22264329 PMC3328258

[B29] Li X Wu HX Dillon SK Southerton SG Generation and analysis of expressed sequence tags from six developing xylem libraries in *Pinus radiata* D. Don BMC Genomics 2009 10 41 10.1186/1471-2164-10-41 19159482 PMC2636829

[B30] Li X Wu HX Southerton SG Transcriptome profiling of *Pinus radiata* juvenile wood with contrasting stiffness identifies putative candidate genes involved in microfibril orientation and cell wall mechanics BMC Genomics 2011 12 480 10.1186/1471-2164-12-480 21962175 PMC3224210

[B31] Li X Wu HX Southerton SG Seasonal reorganization of the xylem transcriptome at different tree ages reveals novel insights into wood formation in Pinus radiata New Phytol 2010 187 3 764 776 10.1111/j.1469-8137.2010.03333.x 20561208

[B32] Allona I Quinn M Shoop E Swope K St CS Carlis J Riedl J Retzel E Campbell MM Sederoff R Analysis of xylem formation in pine by cDNA sequencing Proc Natl Acad Sci U S A 1998 95 16 9693 9698 10.1073/pnas.95.16.9693 9689143 PMC21401

[B33] Kirst M Johnson AF Baucom C Ulrich E Hubbard K Staggs R Paule C Retzel E Whetten R Sederoff R Apparent homology of expressed genes from wood-forming tissues of loblolly pine (*Pinus taeda* L.) with *Arabidopsis thaliana* Proc Natl Acad Sci U S A 2003 100 12 7383 7388 10.1073/pnas.1132171100 12771380 PMC165884

[B34] Pavy N Laroche J Bousquet J Mackay J Large-scale statistical analysis of secondary xylem ESTs in pine Plant Mol Biol 2005 57 2 203 224 10.1007/s11103-004-6969-7 15821878

[B35] Lorenz WW Sun F Liang C Kolychev D Wang H Zhao X Cordonnier-Pratt MM Pratt LH Dean JF Water stress-responsive genes in loblolly pine (*Pinus taeda*) roots identified by analyses of expressed sequence tag libraries Tree Physiol 2006 26 1 1 16 10.1093/treephys/26.1.1 16203709

[B36] Jhanwar S Priya P Garg R Parida SK Tyagi AK Jain M Transcriptome sequencing of wild chickpea as a rich resource for marker development Plant Biotechnol J 2012 10 6 690 702 10.1111/j.1467-7652.2012.00712.x 22672127

[B37] Edwards CE Parchman TL Weekley CW Assembly, gene annotation and marker development using 454 floral transcriptome sequences in *Ziziphus celata* (Rhamnaceae), a highly endangered, Florida endemic plant DNA Res 2012 19 1 1 9 10.1093/dnares/dsr037 22039173 PMC3276261

[B38] Wall PK Leebens-Mack J Chanderbali AS Barakat A Wolcott E Liang H Landherr L Tomsho LP Hu Y Carlson JE Comparison of next generation sequencing technologies for transcriptome characterization BMC Genomics 2009 10 347 10.1186/1471-2164-10-347 19646272 PMC2907694

[B39] Ellis JR Burke JM EST-SSRs as a resource for population genetic analyses Heredity (Edinb) 2007 99 2 125 132 10.1038/sj.hdy.6801001 17519965

[B40] Barbara T Palma-Silva C Paggi GM Bered F Fay MF Lexer C Cross-species transfer of nuclear microsatellite markers: potential and limitations Mol Ecol 2007 16 18 3759 3767 10.1111/j.1365-294X.2007.03439.x 17850543

[B41] Bouck A Vision T The molecular ecologist's guide to expressed sequence tags Mol Ecol 2007 16 5 907 924 17305850 10.1111/j.1365-294X.2006.03195.x

[B42] Rowland LJ Alkharouf N Darwish O Ogden EL Polashock JJ Bassil NV Main D Generation and analysis of blueberry transcriptome sequences from leaves, developing fruit, and flower buds from cold acclimation through deacclimation BMC Plant Biol 2012 12 1 46 10.1186/1471-2229-12-46 22471859 PMC3378433

[B43] Blanca J Canizares J Roig C Ziarsolo P Nuez F Pico B Transcriptome characterization and high throughput SSRs and SNPs discovery in *Cucurbita pepo* (Cucurbitaceae) BMC Genomics 2011 12 104 10.1186/1471-2164-12-104 21310031 PMC3049757

[B44] Zhao Z Boerwinkle E Neighboring-nucleotide effects on single nucleotide polymorphisms: a study of 2.6 million polymorphisms across the human genome Genome Res 2002 12 11 1679 1686 10.1101/gr.287302 12421754 PMC187558

[B45] Keller I Bensasson D Nichols RA Transition-transversion bias is not universal: a counter example from grasshopper pseudogenes PLoS Genet 2007 3 2 e22 10.1371/journal.pgen.0030022 17274688 PMC1790724

[B46] Clark AG Eisen MB Smith DR Bergman CM Oliver B Markow TA Kaufman TC Kellis M Gelbart W Iyer VN Evolution of genes and genomes on the *Drosophila* phylogeny Nature 2007 450 7167 203 218 10.1038/nature06341 17994087

[B47] Kunstner A Wolf JB Backstrom N Whitney O Balakrishnan CN Day L Edwards SV Janes DE Schlinger BA Wilson RK Comparative genomics based on massive parallel transcriptome sequencing reveals patterns of substitution and selection across 10 bird species Mol Ecol 2010 19 Suppl 1 266 276 20331785 10.1111/j.1365-294X.2009.04487.x PMC2904817

[B48] Zakas C Schult N McHugh D Jones KL Wares JP Transcriptome analysis and SNP development can resolve population differentiation of *Streblospio benedicti*, a developmentally dimorphic marine annelid PLoS One 2012 7 2 e31613 10.1371/journal.pone.0031613 22359608 PMC3281091

[B49] Meusemann K von Reumont BM Simon S Roeding F Strauss S Kuck P Ebersberger I Walzl M Pass G Breuers S A phylogenomic approach to resolve the arthropod tree of life Mol Biol Evol 2010 27 11 2451 2464 10.1093/molbev/msq130 20534705

[B50] Roeding F Borner J Kube M Klages S Reinhardt R Burmester T A 454 sequencing approach for large scale phylogenomic analysis of the common emperor scorpion (*Pandinus imperator)* Mol Phylogenet Evol 2009 53 3 826 834 10.1016/j.ympev.2009.08.014 19695333

[B51] Roeding F Hagner-Holler S Ruhberg H Ebersberger I von Haeseler A Kube M Reinhardt R Burmester T EST sequencing of *Onychophora* and phylogenomic analysis of *Metazoa* Mol Phylogenet Evol 2007 45 3 942 951 10.1016/j.ympev.2007.09.002 17933557

[B52] Pal C Papp B Lercher MJ An integrated view of protein evolution Nat Rev Genet 2006 7 5 337 348 10.1038/nrg1838 16619049

[B53] McInerney JO The causes of protein evolutionary rate variation Trends Ecol Evol 2006 21 5 230 232 10.1016/j.tree.2006.03.008 16697908

[B54] Rocha EP The quest for the universals of protein evolution Trends Genet 2006 22 8 412 416 10.1016/j.tig.2006.06.004 16808987

[B55] Wall DP Hirsh AE Fraser HB Kumm J Giaever G Eisen MB Feldman MW Functional genomic analysis of the rates of protein evolution Proc Natl Acad Sci U S A 2005 102 15 5483 5488 10.1073/pnas.0501761102 15800036 PMC555735

[B56] Garzon-Martinez GA Zhu I Landsman D Barrero LS Marino-Ramirez L The *Physalis peruviana* leaf transcriptome: assembly, annotation and gene model prediction BMC Genomics 2012 13 1 151 10.1186/1471-2164-13-151 22533342 PMC3488962

[B57] Li S Chou HH LUCY2: an interactive DNA sequence quality trimming and vector removal tool Bioinformatics 2004 20 16 2865 2866 10.1093/bioinformatics/bth302 15130926

[B58] Kumar S Blaxter ML Comparing de novo assemblers for 454 transcriptome data BMC Genomics 2010 11 571 10.1186/1471-2164-11-571 20950480 PMC3091720

[B59] Huang Y Niu B Gao Y Fu L Li W CD-HIT Suite: a web server for clustering and comparing biological sequences Bioinformatics 2010 26 5 680 682 10.1093/bioinformatics/btq003 20053844 PMC2828112

[B60] Li W Godzik A Cd-hit: a fast program for clustering and comparing large sets of protein or nucleotide sequences Bioinformatics 2006 22 13 1658 1659 10.1093/bioinformatics/btl158 16731699

[B61] van Oeveren J Janssen A Mining SNPs from DNA sequence data; computational approaches to SNP discovery and analysis Methods Mol Biol 2009 578 73 91 10.1007/978-1-60327-411-1_4 19768587

[B62] Milano I Babbucci M Panitz F Ogden R Nielsen RO Taylor MI Helyar SJ Carvalho GR Espineira M Atanassova M Novel tools for conservation genomics: comparing two high-throughput approaches for SNP discovery in the transcriptome of the European hake PLoS One 2011 6 11 e28008 10.1371/journal.pone.0028008 22132191 PMC3222667

[B63] Edgar RC Search and clustering orders of magnitude faster than BLAST Bioinformatics 2010 26 19 2460 2461 10.1093/bioinformatics/btq461 20709691

[B64] Blanc G Wolfe KH Widespread paleopolyploidy in model plant species inferred from age distributions of duplicate genes Plant Cell 2004 16 7 1667 1678 10.1105/tpc.021345 15208399 PMC514152

[B65] Yang Z PAML 4: phylogenetic analysis by maximum likelihood Mol Biol Evol 2007 24 8 1586 1591 10.1093/molbev/msm088 17483113

[B66] Tamura K Dudley J Nei M Kumar S MEGA4: molecular evolutionary genetics analysis (MEGA) software version 4.0 Mol Biol Evol 2007 24 8 1596 1599 10.1093/molbev/msm092 17488738

[B67] Wan LC Zhang H Lu S Zhang L Qiu Z Zhao Y Zeng QY Lin J Transcriptome-wide identification and characterization of miRNAs from *Pinus densata* BMC Genomics 2012 13 132 10.1186/1471-2164-13-132 22480283 PMC3347991

